# Supplementary material for: Identification of potential riboswitch elements in Homo sapiens mRNA 5’UTR sequences using positive-unlabeled machine learning
Source: PLoS One. 2025 Apr 24;20(4):e0320282. doi: 10.1371/journal.pone.0320282 (PMC12021280; doi:10.1371/journal.pone.0320282)
Supplement: Attachment — Submitted filename: Response_to_reviewers_comments.pdf [file pone.0320282.s004.pdf]

Response to reviewer comments.

Below we reproduce the reviewer comments in their entirety (italics) followed by our responses (in blue) from our submission to *PLoS Computational Biology* (Manuscript number: PCOMPBIOL-D-23-01896, editor: Marcel Holger Schulz, Ph.D.)

Reviewer #1*: In this paper, the authors utilize a positive-unlabeled machine learning-based approach to identify potential riboswitches in human 5’ UTR sequences. While the method described is novel for riboswitch-based sequence screening approach, the results presented are insufficient to conclusively support the identification of these sequences as potential human riboswitches.*

We thank the referee for their consideration of our manuscript. We agree that our presented computational method is novel, and that this is the main strength of the presented manuscript. Unfortunately as a computational group, we are not equipped to conduct the experiments needed to fully validate the discovery of a riboswitch. Instead, we have added additional validation steps drawing on existing experimental data to demonstrate that our approach can reliably identify held out natural and synthetic riboswitches (including a small number that have been discovered in eukaryotes) even when the learning approach has never had access to training data for riboswitches that respond to the same ligands. We further demonstrate that the approach correctly rejects sequences (e.g., random sequences and exons) that are known to be highly unlikely to harbor riboswitches.

Comment 1: *While it's true that bacterial riboswitches are typically located in the 5’ UTR, the TPP riboswitch, found in fungi and plants, is located in intronic regions and the 3’ UTR. This evidence suggests that, throughout evolution, riboswitches have preserved their structure but vary in gene location and regulatory mechanisms. The authors have limited their search to 5’ UTRs without considering structural conservation. A more comprehensive search should include analyzing 5’ UTRs, 3’ UTRs, and intronic sequences to identify potential human riboswitch candidates.*

Our approach and provided codes can be reused for intronic and 3'UTR searching -- however, we have two reasons for using the 5'UTR first: Using the 5'UTR lets us know the most likely location of likely action (around the start codon) for us to perform machine learning on, reducing the need for sequence scanning of the relatively longer 3'UTRs. For introns the location of the potential riboswitches is likely near the splice sites for future searching. Second, only using the 5'UTR ensures a smaller list of sequences to examine via experimentation. The goal of the paper is to start the search, hopefully finding one human riboswitch. Providing experimentalists with a larger list is costly to their resources and risks experimentalists ignoring the list. Once one riboswitch is experimentally validated, this PU ensemble approach can be easily replicated again on the other two regions of interest.

Additionally, as suggested by the referee, we are indeed using features taken from the predicted secondary structure of the RNA sequences, we are using this information as a proxy for structural information for the classifiers to learn on. We added a supplemental figure that shows that indeed the most informative features are the counts of these structural features. In other words, while we aren’t looking for exact conservation, our machine learning can still learn structural trends and is identifying them as the most important features.

Comment 2: *Functional riboswitches should exhibit two structural states. Therefore, in identifying new riboswitch candidates, it is crucial to consider whether a given RNA sequence can adopt two mutually exclusive folding conformations. Analyzing the folding landscape of the predicted riboswitch sequence and identifying key bifurcation states is essential. Without this consideration, the process of identification is less meaningful.*

This is an excellent suggestion. We have now added the NUPACK base pair probability charts from 1000 simulated foldings to the display website for each predicted hit. Additionally, we break down this probability chart into conformers where the start codon is bonded or unbonded (providing an additional justification for our focus on the 5'UTR). We have not updated our feature selection approach to include conformer information; Future machine learning efforts could use the base pair probability matrix instead of structural information – however, we believe our cross-validation on with-held riboswitches that our current approach is sufficient to justify that our approach correctly selects for riboswitches. Below is an example of these probability charts that shows two distinct conformers that either block (blue) or reveal (red) the start codon in the merge chart (bottom right of figure below).

NUPACK base pair probabilities for the 5'UTR of the human gene RORA

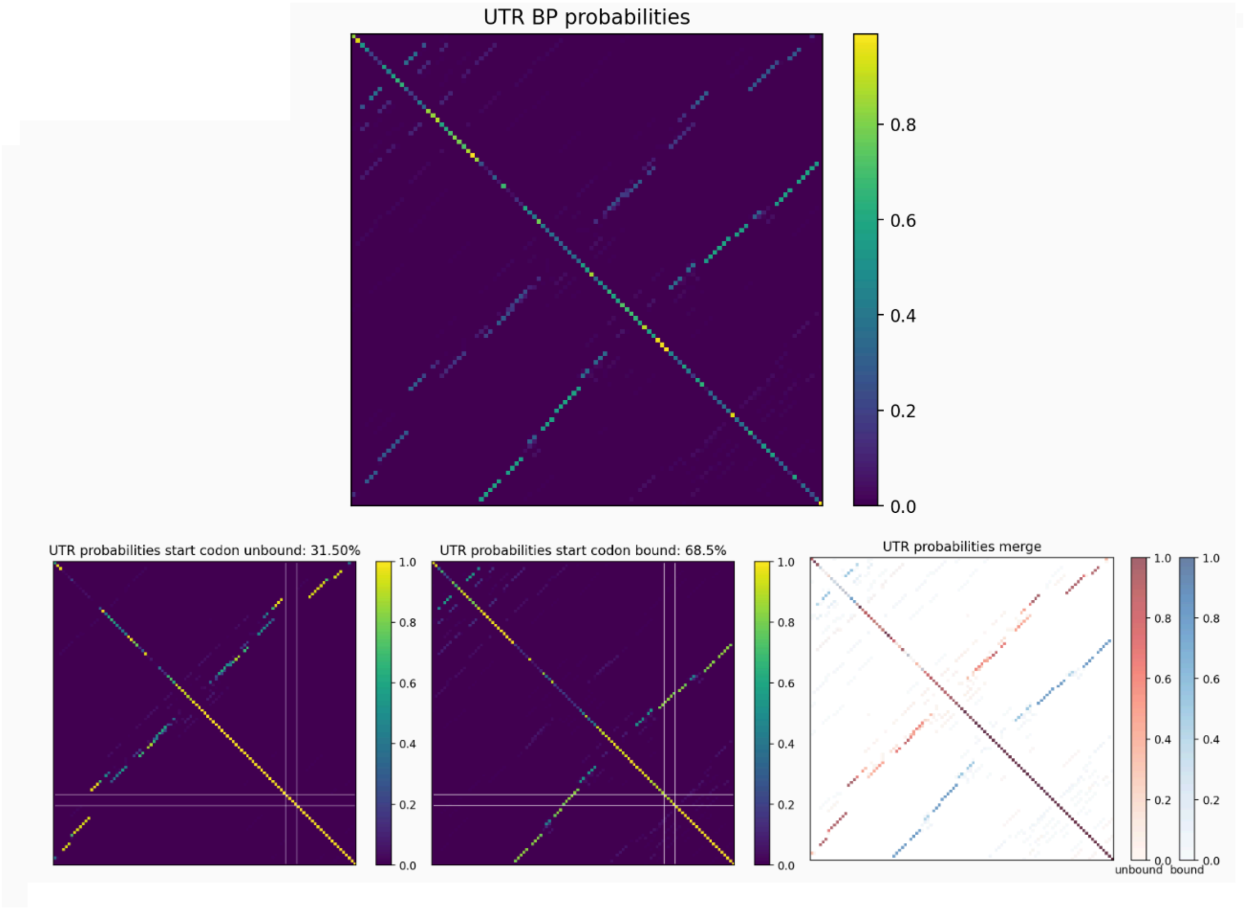

Comment 3: *While Gene Ontology (GO) analysis provides a broad overview, it fails to offer specific insights into the riboswitch function. The authors were unable to functionally classify the riboswitch sequences. A comparative genomic analysis across species is necessary, where the predicted riboswitch sequences should*

be analyzed. By classifying each riboswitch candidate based on orthologs and analyzing the functionality of the associated genes, a more detailed understanding of the functionality of the predicted riboswitch candidates could be achieved.

The authors acknowledge that this approach could identify more relations, however, we believe that such an analysis would be beyond the current scope. The manuscript's primary goal is to provide the list for experimental validation. Ultimately the experimental validation will provide the key findings and relationships as once one riboswitch is discovered, traditional sequence based bioinformatics can take over to extrapolate from our list.

—-----

Reviewer #2: *The topic of the research is of interest: Are there potential riboswitch elements in eukaryotes, or even in Homo Sapiens mRNA 5'UTR? Moreover, state-of-the-art methods are used and the paper is written properly.*

Comment 1: *However, positive unlabeled machine learning is used and we learn: "436 sequences were mapped back to the most similar riboswitches within the positive data and examined. An online database of identified and ranked 5'UTRs, their features, and their most similar matches to known riboswitches, is provided to guide future experimental efforts to identify H. sapiens riboswitches."*

This is not sufficient as the reader does not know anything about the quality of the data. The key is to trust in ones own predictions and to validate at least one of the putative structures by experimental data before claiming that the correct structure was recognized. So this is a must before this can be published in a good journal. If the author itself does not trust the prediction, it is a leap of faith to think somebody else will do then the experimental work to be sure.

The authors acknowledge this limitation of our paper. Our lab does not currently have the capability to perform the experimental validation ourselves. From our perspective, this analysis or a similar one would have to be taken by any experimentalist wishing to work on this problem as they cannot check 60,000+ isoforms of mRNA themselves, there must be some sort of statistical pruning beforehand. Our paper provides an example of such a pruning, which future experimentalists can use as a starting point for their own analyses.

Although we cannot personally collect new data, we do extensive validation against existing data. Our validation uses leave-one-out cross validation on entire structural classes with a very high selection threshold for our predicted hits. This ensures that we are selecting 5'UTR hits with features that are extremely similar to known Riboswitch features. While we cannot fully trust our outputs without experimental data, as noted in the paper, we have added additional negative and positive controls for testing our machine learning ensemble with extremely satisfactory results (See also our detailed response to comment 4 below).

Comment 2: *If this experimental test is missing, how do you think anybody else would start the fishing expedition of experimental testing? It is very time consuming and expensive to test 436 riboswitches in the lab, even more so as may be none of these predictions could perhaps work out in the end.*

We provide a ranking of our hits based on feature similarity and our ensemble prediction. We would prefer that experimentalists use this ranking for their search, going down the list of highest similar or highest ensemble output. As a purely computational lab, we cannot comment on potential experiment designs. However, anyone attempting this problem would have to do some downselection of the total mRNA isoforms in the human translatome, and we believe that the provided computational analysis could assist with this process similar to how theory and mathematical models have driven discovery in the physical sciences.

Comment 3: *Moreover, machine learning is a black box, it does not explain which features it exactly recognized.*

The authors acknowledge that statistical machine learning methods are often presented as black boxes, but it is possible to gather some insight into the statistical workings of the ML classifier. To address this concern, we have added a supplemental figure that explores the discrimination importance for each individual feature under consideration. We find that the most informative features are the enumeration of structural features and not the sequences themselves.

Moreover, the training was done on other species, mostly bacteria -- this is bound to fail on other species, particular if eukaryotes are used and even more so if the human species is tried out.

Although the referee's concern that variations between organisms will reduce the ability to classify based on RNA sequence is valid, we note that cross-domain approaches such as the one taken in Mukherjee et al 2023 have shown that structures may be conserved even if sequences change significantly. Specifically, the example of a purine riboswitch in fungi was found through structure conservation of a bacterial riboswitch. By defining our approach to classify based on both structural and sequence based information, we are hoping to make use of similar structural information, and this was a large part of the motivation for our analysis approach.

Moreover, the cross-species problem is addressed through our multiple applications of cross validation. In our validation studies, the classifiers are tested for their ability to discover entire withheld riboswitch classes (i.e., riboswitches that respond to the same ligand) that are not represented within their training data. Given the sparse availability of known riboswitches, we acknowledge that both our training data and our validation data are predominantly (but not exclusively) bacteria. In the revised manuscript, we now examine approximately 800 examples of the only known class of eukaryotic riboswitches (TPP), and we find that our ensemble classifies 88.5% of these correctly as riboswitches (that is, we achieved an average of 88.5% across the 20 classifiers that make up the ensemble). Given our success to identify the limited set of known eukaryote TPP riboswitches, even for classifiers that do not have access to *any* TPP riboswitches in their training data, we believe that our results provide a good start toward finding other eukaryotic riboswitches for TPP or other ligands.

Comment 4: *We learn:*
"PU learning and cross validation achieves 75% to 99% accuracy to identify held out known riboswitches."
So does this mean, in a quarter of the cases even the positive control was not recognized?

Sure, some examples have 99% accuracy, but this shows again: it is completely unclear how well the machine learning performs here if a specific structure is analyzed. It would also be good to know more about false positives, e.g. was human ribosomal RNA or other RNA also classified as riboswitch though we know for sure that it has NO riboswitch?

Of the 20 PU learning classifiers we examined, their ***validation*** accuracy (middle ring below) ranges from 75% to 99% (median is 90.5%), meaning this is how each individual classifier performs on a particular ligand class, when that entire class was withheld from training. The training accuracy is much higher (outer ring below, 95-97%). The validation process is simulating how a given classifier performs on an unknown riboswitch class, while it learns on all the other known examples. Given this consideration, we believe that 75-99% accuracy on a set of riboswitches a classifier has not encountered is quite good. The range in the validation accuracies (Figure 3 within the paper) is due to the particular ligand's riboswitches and their representation and similarity to the other riboswitch classes.

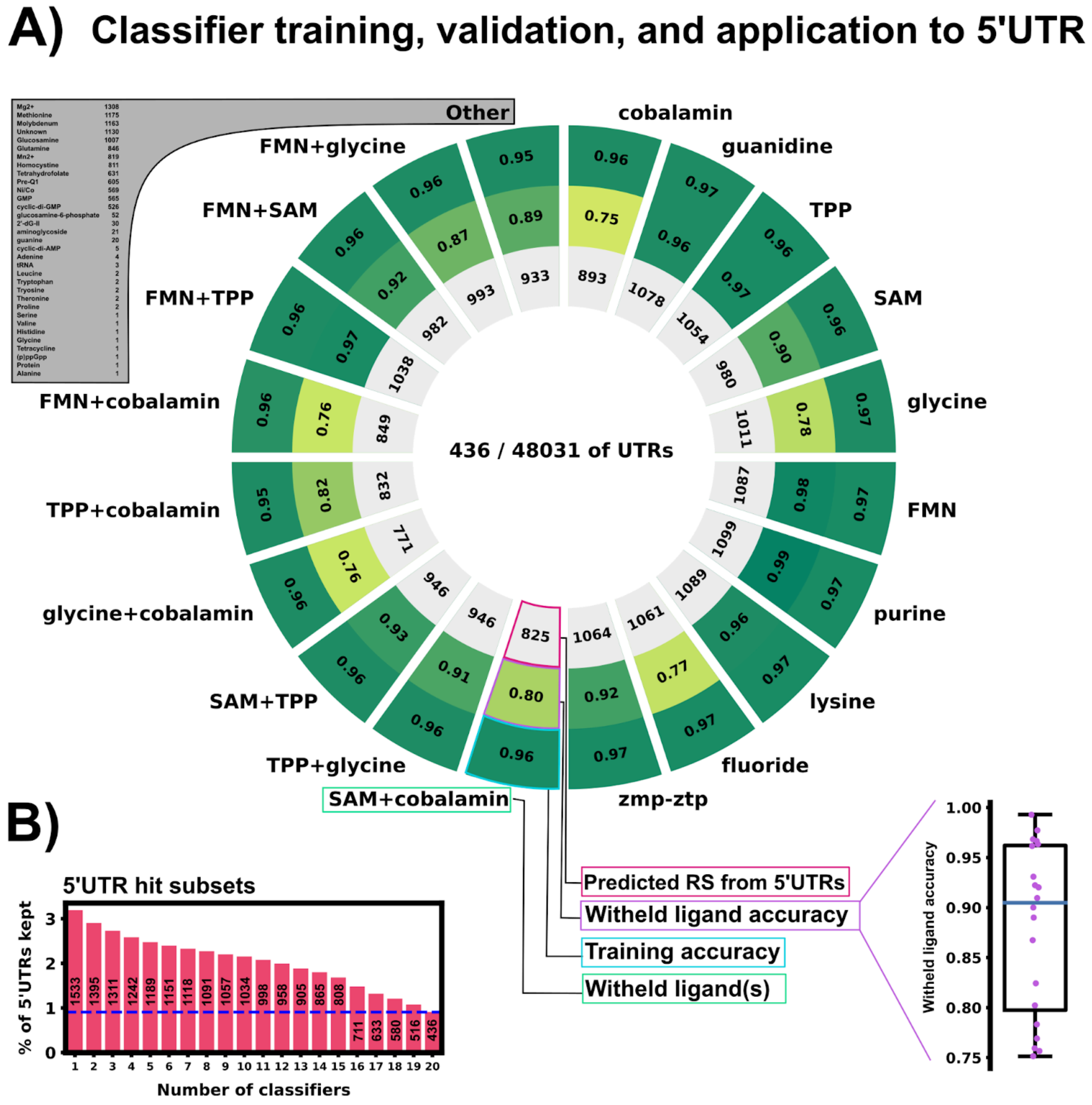

As for the second comment, we acknowledge a limitation in our ability to estimate a false positive rate – the true class prevalence of riboswitches within the 5'UTR can only be determined via extensive experimental validation, a common issue within PU learning in general. We suggest the desired order in which to experimentally test our hits, but we cannot perform the tests ourselves. Despite this limitation, we have added an approximate false positive rate analysis showing a new ensemble's output when including "junk" sequences as suggested: i.e, for both random and human exons. Our ensemble training approach soundly rejects all the random and exonic sequences with a low false positive rate when compared to "kept" 5'UTRs.

Ensemble results when training with Exon and Random seqeuences

	Riboswitch	5'UTR	Exon	Random	
N total	67683	48031	5946	60000	
average ensemble % labeled RS	95.033%	7.081%	0.146%	0.019%	P(seq) > 0.5
N found	64320	3400	8	11	
average ensemble % labeled RS	82.037%	2.044%	0.020%	0.000%	P(seq) > 0.95
N found	55525	981	1	0	
	positives	positives + false positives	false positives	false positives	

overlap with original ensemble: 392/436

We have taken care in the writing to note that this FPR analysis doesn't give an idea of the true prevalence of riboswitches within the 5'UTR, but rather a confidence in our feature selection and machine learning approach.

Comment 5: *In summary, this is preliminary work, only the first part of a study, but experimental proof, critical in this case, is lacking. Moreover, using an intransparent method like machine learning does not inspire confidence in the list generated and yes, there are may be some riboswitches in this list, but as everything was trained on other species and even organisms (mostly bacteria) the ranking achieved is completely doubtful. An experimentalist would really have to screen all 436 sequences to find out what the listing really achieves.*

Yes, we agree that this work is preliminary in nature as far as experimental validation goes, but we believe that we have performed due diligence in verifying that the approach can accurately rediscover known riboswitches in the literature. As discussed above, we have applied cross validation to verify that the approach correctly enriches for unseen riboswitches (i.e., even when the model has never seen similar riboswitches that respond to the same ligand) as well as synthetic riboswitches and a handful of rare riboswitches that have been found in eukaryotes. Moreover, the model rejects random sequences and exonic sequences that are expected not to harbor riboswitches. As a result, the presented analyses reduce the set of possible isoforms (60000+) to a more manageable ranked set of 436. Furthermore, we provide a clear set of customizable codes so that future experimentalists can perform similar structure based searches with additional training data or structural features should such data become available in the future.

Comment 6: *The solution to push your study further would be to zoom in on the only one riboswitch class really known to work in eukaryotes. For instance, start with this article*
https://www.sciencedirect.com/science/article/pii/S0968000422002341
"Discovering riboswitches: the past and the future" (Kavita et al., review 2021 in TIBS)
and there you find:

"Of all the bacterial riboswitch classes that have been experimentally validated over the past two decades, only one class has convincingly been shown to function naturally in eukaryotic species. TPP riboswitches [2,3], which are the most abundant single class in bacteria [7], are also relatively common in fungi and plants [91., 92., 93.]."

So, best one first starts with the only experimentally validated known riboswitch in eukaryotes, the TPP riboswitch, confirmed in fungi and plants, and find some new putative instances with your method in other fungi or plant species, but now trained exactly on the TPP riboswitch and then include experimental validation of your top candidates.
Once you have achieved that, well, then start looking is a TPP riboswitch also in vertrebrates and may be even in humans, but you need again experimental proof in one species and then you can look with your method into a related species, but check carefully negative and positive controls and detail them in your manuscript.

The authors acknowledge we could have limited our machine learning training to one type of riboswitch – this may have resulted in a more accurate, but specialized TPP-riboswitch model. However, this would severely limit the generalizability of the approach because (1) there would be a much smaller amount of training data if we ignored all other riboswitch classes, and (2) the resulting model would be unlikely to detect non-TPP riboswitches. Additionally, (3), we would lose our structural cross validation approach if we only had one ligand to consider, especially a ligand with limited structural diversity.

Instead, we decided to train the model under the consideration of all known riboswitches and show that this model would have been able to discover TPP (and all other ligand-responsive) riboswitches without prior knowledge that such riboswitches exist. The ability for the resulting model to discover TPP riboswitches (including the smaller subset of TPP riboswitches that are in eukaryotes) adds confidence that the approach could detect riboswitches that respond to other, hopefully undescribed, ligands. Although we have not focused on TPP-riboswitches, the provided website and Github allows for experimentalists to limit their search to only predicted similar TPP riboswitches.
