# Supplementary material for: Identification of potential riboswitch elements in Homo sapiens mRNA 5’UTR sequences using positive-unlabeled machine learning
Source: PLoS One. 2025 Apr 24;20(4):e0320282. doi: 10.1371/journal.pone.0320282 (PMC12021280; doi:10.1371/journal.pone.0320282)
Supplement: Attachment — Submitted filename: Response to Reviewers.pdf [file pone.0320282.s005.pdf]

Dear PLOS ONE editors and manuscript reviewers,

We would like to express our gratitude for the editors and reviewers' time. We have endeavoured to address all the comments raised about formatting from the editors and comments on content from the reviewers as well as some small general corrections. Below is our response to each numbered point in the decision letter.

Response to editors

1. The authors have checked the formatting style guidelines for PLOS ONEand ensured that their manuscript adheres to these requirements.
2. We have checked that our code meets the requirements of PLOS ONE's open source publishing.
3. We have removed the following funding statement from the manuscript: "WSR, and BM were supported by the NSF (1941870) and National Institutes of Health (R35GM124747). JD was supported by the National Institutes of Health (1R01AI168459-01A1)." We have moved this statement to the cover letter as requested.
4. We have reviewed author affiliations and made no changes
5. The authors have reviewed our cited references, one reference (22, riboflow) was fixed from referencing a preprint to its new publishing location. Several DOI links were corrected as they had extraneous links to specific parts of manuscripts. Now these refer to their manuscript.

Reviewer comments

> Reviewer #2: The authors have satisfactorily addressed the previous reviewers comments. I have one additional comment to the authors.
> • This study on identifying potential riboswitch elements in human mRNA 5'UTR sequences provides valuable insights. To enhance the context of the findings, I recommend citing the article "Regulation of mRNA translation by a photoriboswitch". (https://elifesciences.org/articles/51737) This work discusses how riboswitches, including photoriboswitches, regulate mRNA translation in response to light, offering valuable insights into the functional roles of riboswitches. Citing this paper would further highlight the potential applications of the riboswitch elements identified by the authors, particularly in controlling gene expression.

The authors agree that this paper has merit, and synthetic riboswitch uses in synthetic biology should be mentioned in the manuscript. We have added the following sentence to the introduction to address this comment:

"Besides being a critical regulatory tool in nature, both natural and synthetic riboswitches have been used in synthetic biology for responsive translational control, splicing control, and photo-regulation~\cite{Etzel2017,Kelvin2023,Rotstan2020}"

Additional changes

We have fixed an error in the introduction where we say our ensemble's accuracy on withheld synthetic riboswitches was 56%. This does not reflect the actual section in results where we report an 84% accuracy. This discrepancy between sections is due to a previous coding bug. An earlier version of the manuscript reported an accuracy of 56% due to one structural feature input

being incorrectly normalized. When this was fixed, classification jumped from 56% to 84% at a 0.95 threshold and we neglected to correct the introduction.

Additionally, we have changed the following sentence: “...correctly discovered as riboswitches despite having no representation of similar synthetic riboswitches in any training data.” to “...correctly discovered as riboswitches despite having minimal representation of similar synthetic riboswitches in any training data.” This change is to reflect that a tiny percent (14/~60k) of synthetic switches are included in the training data, even though they aren’t the 25 theophylline switches used for validation.

Please let us know if there are any additional requirements from us, and we would like to thank the reviewers and editors again for their time.

Sincerely,

Dr. William Raymond
School of Biomedical Engineering
Colorado State University
